# T cell receptor (TCR)-transgenic CD8 lymphocytes rendered insensitive to transforming growth factor beta (TGFβ) signaling mediate superior tumor regression in an animal model of adoptive cell therapy

**DOI:** 10.1186/1479-5876-10-127

**Published:** 2012-06-19

**Authors:** Jon G Quatromoni, Yue Wang, Dan D Vo, Lilah F Morris, Ali R Jazirehi, William McBride, Talal Chatila, Richard C Koya, James S Economou

**Affiliations:** 1 Departments of Surgery, University of California, Los Angeles, CA 90095, USA; 2 Radiation Oncology, University of California, Los Angeles, CA 90095, USA; 3 Pediatrics, § Microbiology, Immunology and Molecular Genetics, University of California, Los Angeles, CA 90095, USA; 4 Molecular and Medical Pharmacology, University of California, Los Angeles, CA 90095, USA; 5 Jonsson Comprehensive Cancer Center, David Geffen School of Medicine, University of California, Los Angeles, CA 90095, USA; 6 Division of Surgical Oncology, UCLA David Geffen School of Medicine, 10833 LeConte Ave, 54–140 CHS, Box 957182, Los Angeles, CA 90095-1782, USA

## Abstract

Tumor antigen-reactive T cells must enter into an immunosuppressive tumor microenvironment, continue to produce cytokine and deliver apoptotic death signals to affect tumor regression. Many tumors produce transforming growth factor beta (TGFβ), which inhibits T cell activation, proliferation and cytotoxicity. In a murine model of adoptive cell therapy, we demonstrate that transgenic Pmel-1 CD8 T cells, rendered insensitive to TGFβ by transduction with a TGFβ dominant negative receptor II (DN), were more effective in mediating regression of established B16 melanoma. Smaller numbers of DN Pmel-1 T cells effectively mediated tumor regression and retained the ability to produce interferon-γ in the tumor microenvironment. These results support efforts to incorporate this DN receptor in clinical trials of adoptive cell therapy for cancer.

## Background

Metastatic melanoma continues to be a therapeutic challenge. Adoptive cell therapy (ACT) using tumor-infiltrating lymphocytes (TILs) or T cell receptor-engineered lymphocytes has produced increased response rates, many clinically dramatic, but most are partial and patients generally relapse within a short time frame [1-3]. Factors contributing to these partial responses include down-regulation of MHC and antigen presentation by tumor cells, their resistance to T cell delivered death signals, and tumor production of immunosuppressive factors such as vascular endothelial growth factor (VEGF), Indoleamine-pyrrole 2,3-dioxygenase (IDO), IL10 and transforming growth factor-beta (TGFβ) [4,5].

Many reports have shown that melanoma and other cancers produce TGFβ which in turn promotes tumor invasion, metastasis and creates an immunosuppressive microenvironment that inhibits immune effector function [6,7]. TGFβ inhibits T cell activation, proliferation, cytotoxicity and promotes T regulatory cell inhibitory functions [8-11].

Different strategies to circumvent the inhibitory effects of TGFβ have been employed with varying success. The use of a dominant-negative TGFβ receptor II (DN) as a decoy receptor to render T cells insensitive to TGFβ signaling allows them to retain proliferative and cytotoxic functions in the presence of exogenous TGFβ and to acquire resistance to inhibition by T regulatory cells (Treg) [12-15]. Transgenic mice with TGFβ insensitive T cells are resistant to lymphoma and melanoma tumor challenge [16].

In this study, we used an animal model of ACT in which antigen-reactive T cells were rendered insensitive to TGFβ through transduction with a DN TGFβ RII transgene. These TGFβ-insensitive, antigen-specific T cells mount a more effective anti-tumor response towards B16 melanoma, most likely due to the ability of these effector cells to remain active in the tumor microenvironment.

## Materials and methods

### Mice and cell lines

C57BL/6 (The Jackson Laboratory) and Pmel-1 (kind gift from Dr. Nicholas Restifo, Surgery Branch, National Cancer Institute) [17] mice were bred and kept under defined-flora pathogen-free conditions at the American Association for Laboratory Animal Care–approved Animal Facility of the Division of Experimental Radiation Oncology, University of California, Los Angeles. The B16 murine melanoma cell line (American Type Culture Collection) was maintained in DMEM (Mediatech) with 10% FCS (Omega Scientific) and 1% (v/v) penicillin, streptomycin, and amphotericin (Omega Scientific).

### Western blot analysis for p-SMAD2 expression

Pmel-1 T cells were transduced with DN TGFβ RII (DN) retrovirus and sorted by FACS to ensure >98% TGFβ RII expression. Untransduced and DN-transduced pmel-1 cells were incubated in 10 ng/ml TGFβ for 0, 0.5, 1, 6, 24 h prior to protein extraction. Cells were lysed at 4°C in radioimmuno-precipitation assay (RIPA) buffer [50 mM Tris–HCl (pH 7.4), 1% NP-40, 0.25% sodium deoxycholate, 150 mM NaCl] supplemented with one tablet of protease inhibitor cocktail (Complete Mini; Roche) and phosphatase inhibitors (Santa Cruz Biotechnology, Santa Cruz, CA). A detergent-compatible protein assay kit (Bio-Rad, Hercules, CA) was used to determine protein concentration. An aliquot of total protein lysate was diluted in an equal volume of 2X SDS sample buffer, boiled for 5 min, and cell lysates were electrophoresed on 10% SDS-PAGE gels. Western blot was carried out with anti-phospho-Smad 3 antibody (Cell Signaling Technology, Danvers, MA) at 1:500 overnight at 4 C, anti-total-Smad 3 antibody (Cell Signaling) at 1:1000 overnight at 4 C, and anti-β-Actin-HRP antibody (Sigma, St. Louis, MO) at 1:20,000 for 2 h at room temperature. Secondary anti-rabbit-HRP (Santa Cruz) was used at 1:10,000 for 30 min.

### Retrovirus production and transduction of pmel-1 splenocytes

The vector containing the dominant negative TGFβ receptor II, which encodes a truncated receptor lacking the entire kinase domain and most of juxta-membrane region was constructed by PCR amplification, using the TGFβ receptor II cDNA as template [18]. Primers were designed to include a stop codon after nucleotide 597, which corresponds to the 10th cytoplasmic codon region, flanked by Sal I and Bam HI sites. Amplified fragment was then inserted into the retroviral vector MSCV. DN TGFβ RII retrovirus was produced as previously described [19]. T cells were transduced 48 hours following polyclonal activation with anti-CD3 and anti-CD28 (BD Bioscience) coated plates. 2.5 × 10^6^ cells per well were transduced in 3 ml total volume of virus and culture media in retronectin coated 24-well plates. Cells were spinnoculated twice by centrifugation at 1000 × *g* for 2 hr at 32°C.

### Bone marrow–derived dendritic cells

The generation of dendritic cells from murine bone marrow in cultures of granulocyte-macrophage colony-stimulating factor (GM-CSF) and interleukin (IL)-4 was described previously [20]. Day 8 dendritic cell cultures were pulsed for 2 h with 1 μg/ml gp100_25-33_ peptide for the Pmel-1 adoptive transfer model. Between 5 × 10^5^ and 1 × 10^6^ cells per mouse were injected s.c. in the right flank.

### Pmel-1 adoptive transfer therapy *in vivo* model

B16 tumors were implanted s.c. as described previously [20]. When tumors reached 5 to 8 mm in diameter, mice received a myeloablating regimen of 900 cGy total body irradiation. The following day, Pmel-1 splenocytes were adoptively transferred into 8 experimental mice per group via a lateral tail vein. Subsequently, gp100_25-33_ peptide-pulsed dendritic cells were given s.c. on the day of adoptive transfer and 1 week later, in both cases, followed by 3 days of daily i.p. administration of 50,000 IU IL-2. For co-adoptive transfer model, mock and DN-transduced Pmel-1 were added and mixed at 1:1 ratio prior to adoptive transfer of 10^6^ activated Pmel-1 followed by two rounds of IL-2 administration.

### Flow cytometry analysis

Splenocytes and tumor-infiltrating lymphocytes, obtained from enzymatically digested B16 tumors harvested from mice as described previously [21], were stained with antibodies to CD8α^FITC^, TGFβ RII^PE^, Thy1.1^PerCP^, and CD3^APC-Cy7^ (BD Bioscience), and analyzed with a FACSCalibur machine using FCS Express software (DeNovo Software). Cells were initially gated on live cells area by FSC x SSC analysis, then gated the CD3 positive/CD8 positive/Thy1.1 positive (staining for pmel-1 T cells), followed by TGFβ RII levels analysis. Intracellular IFN-γ staining was done as described previously [22]. Briefly, 1million cells were stimulated with 1 μM specific peptide (gp100_(25–33)_) or non-relevant peptide Ovalbumin, plus brefeldin A (BD Pharmingen) and 50 U/ml IL-2, for six hours at 37°C in 5% CO2. Cells were then washed with staining buffer (PBS with 3% FBS and 0.09% sodium azide), pre-treated with anti-FcR Ab for 10 min, and then stained with anti-CD4, anti-CD8, and anti-Thy1.1 (BD Pharmingen) on ice for 30 min. Cells were then permeabilized and fixed with Cytofix/CytoPerm (BD Pharmingen), then stained for intracellular IFN-γ with anti-IFN-γ or a isotype control mAb.

## Results

### Pmel-1 CD8 T cells can be transduced to high efficiency with a DN TGFβ retrovirus

**Figure 1:**
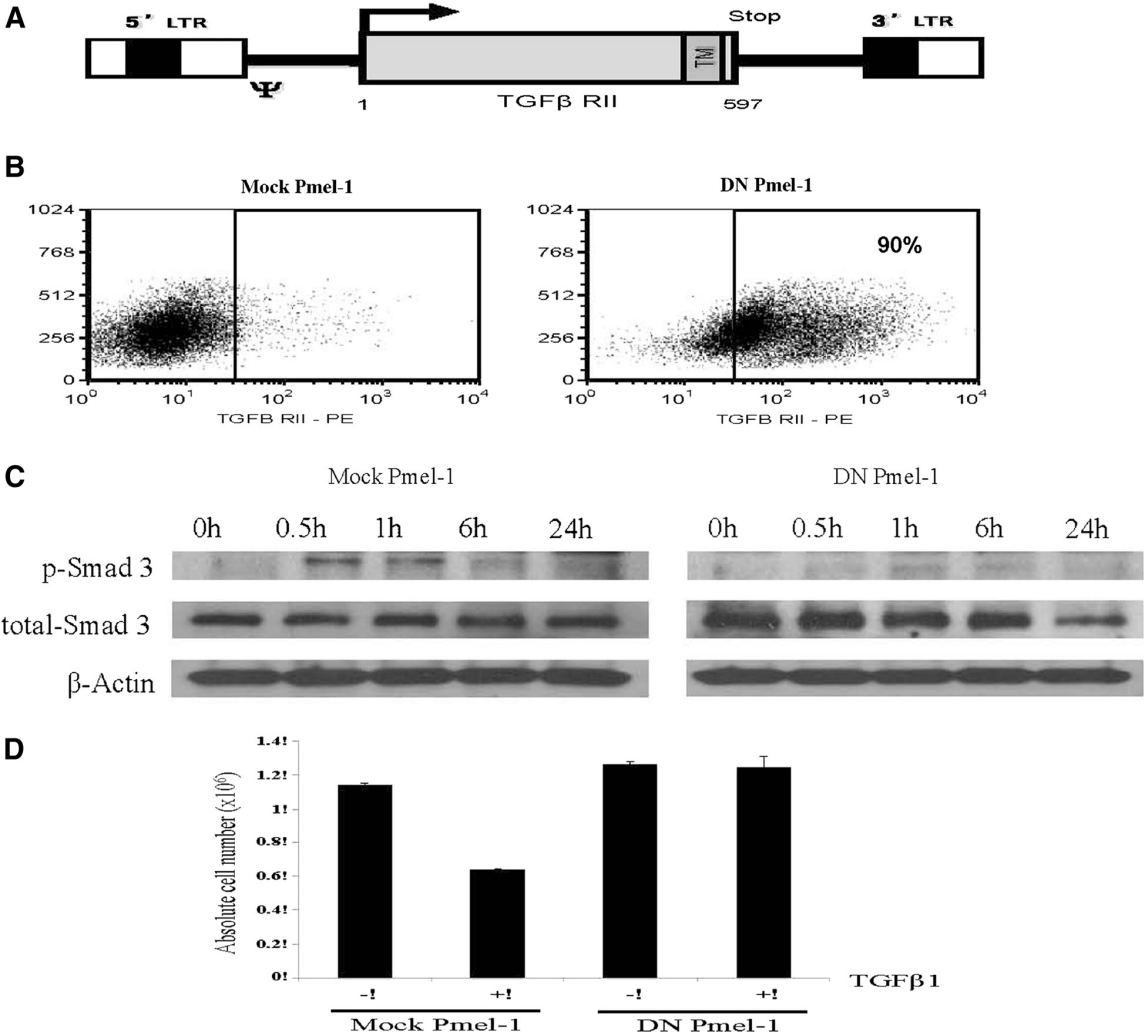
**Pmel-1 CD8+ T cell transduction and expression of DN TGFβ RII.A**. Linear map of DN TGFβ RII retroviral vector, TM = transmembrane region. **B**. Representative dot plot of mock or DN transduced Pmel-1. **C.** Untransduced or DN-transduced Pmel-1 T cells were incubated in 10 ng/ml TGFβ for 0, 0.5, 1, 6, 24 hr prior to protein extraction and analyzed for expression of phosphorylated SMAD3. In the presence of TGFβ, untransduced Pmel-1 T cells demonstrated high expression level of phosphorylated SMAD3 protein at 0.5 and 1 hr whereas DN transduced Pmel-1 T cells did not show p-SMAD3 expression, indicating DN transgene effectively inhibits TGFβ signaling in transduced cells. **D**. Absolute cell counts of Pmel-1 cells re-activated with IL-2 (50 IU/ml) and gp100 peptide (1 μg/ml) and treated with mouse TGFβ1 (10 ng/ml) for 72 h.

The retroviral vector encoding the DN TGFβ RII (DN), in which the intracellular signaling sequence was deleted, is depicted in Figure 1A. Activated Pmel-1 splenocytes can be transduced to high efficiency (70-90%) with this vector. Shown in Figure 1B are DN-transduced and mock-transduced Pmel-1 splenocytes stained with an antibody for the human (not murine) TGFβ RII receptor. The right hand panel (DN Pmel-1) shows the levels of enrichment of human DN receptor transgene after transduction. This DN TGFβ receptor has been shown in previous studies to inhibit TGFβ signaling. Pmel-1 T cells, transduced with the DN receptor, did not phosphorylate SMAD3 after incubation with exogenous TGFβ1 (Figure 1C). The proliferation of mock-transduced, but not DN-transduced, Pmel-1 cells was inhibited after exposure to TGFβ1 (Figure 1D). These results confirm that this DN receptor inhibits the anti-proliferative effects of TGFβ (12).

### DN TGFβ -transduced pmel-1 more effectively mediate B16 tumor regression

**Figure 2:**
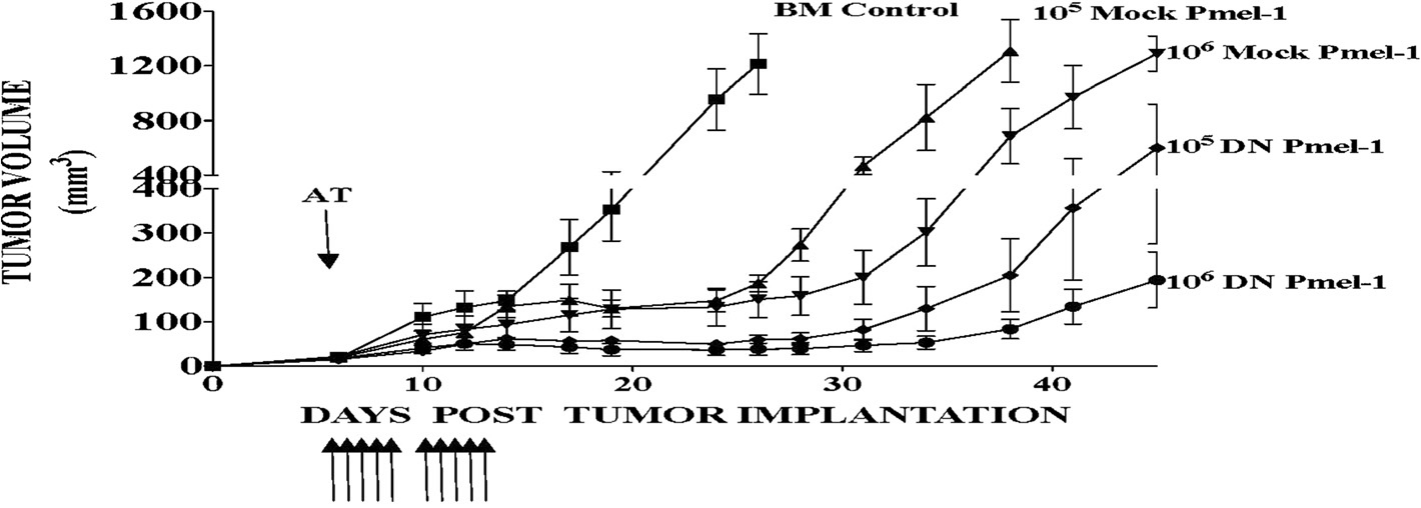
**TGFβ insensitive Pmel-1 cells more effectively mediate B16 tumor regression.** B16 tumor-bearing mice were conditioned with 900 cGy irradiation and given bone marrow support prior to adoptive transfer of either 10^5^ or 10^6^ mock or DN transduced Pmel-1 and IL2 support. Compared to mock transduced Pmel-1, DN Pmel-1 demonstrate better anti-tumor activity. Experiment was performed twice with similar results.

Pmel-1 CD8 splenocytes express a transgenic TCR that recognizes gp100_25-33_ in the context of H-2D^b^; adoptive transfer of activated Pmel-1 can mediate partial or complete regression of established B16 melanoma in various animal tumor models [23,24]. The B16 melanoma used in these experiments produced about 1 μg TGFβ/10^6^ tumor cells/24 hr. C57BL/6 mice bearing small-established B16 tumors (~64 mm^3^) experienced significant delay in tumor outgrowth upon adoptive transfer of activated Pmel-1 T cells (Figure 2). A myeloablative conditioning regimen of 900 cGy whole body irradiation (accompanied by bone marrow rescue) creates space in the secondary lymphoid organs enabling better repopulation by administered T cells. Administration of IL-2 and/or gp100_25-33_ peptide pulsed dendritic cell vaccine (gp100_25-33_/DC) supports the expansion and anti-tumor activity of administered Pmel-1 T cells. Figure 2 depicts a direct comparison between DN-transduced (80% transduction efficiency) and mock transduced Pmel-1; 10^5^ DN Pmel-1 produced greater tumor suppression than 10^6^ mock Pmel-1. These animals were supported by IL-2 administration alone.

### DN TGFβ effector T cell function with IL-2 and/or gp100_25-33_/DC support

**Figure 3:**
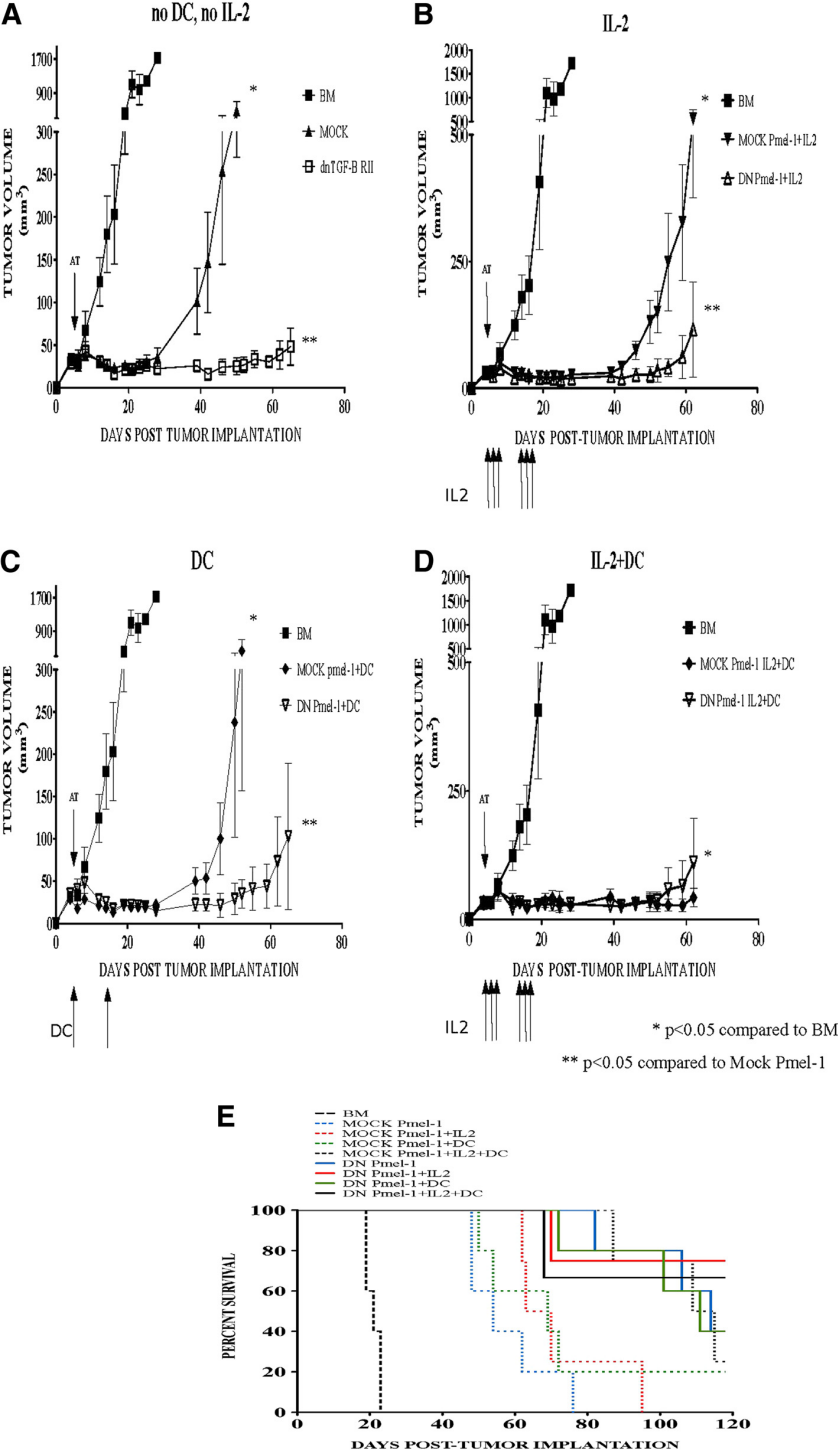
**DN Pmel-1 mediate tumor regression without IL2 and gp100**_**25-33**_**/DC support.** Mice bearing subcutaneous B16 tumors were myeloablated and given BM support prior to receiving adoptive transfer of 10^6^ mock or DN Pmel-1. Subsequently, mice received IL2 or gp100_25-33_/DC support, both, or neither. Even without IL2 and gp100_25-33_/DC support, DN Pmel-1 mounted robust anti-tumor activity, repressing B16 tumor outgrowth up to 90 days post tumor implantation (3**A**). Support with IL2, gp100_25-33_/DC, or both lead to better tumor protection, however, DN Pmel-1 still demonstrate superior anti-tumor activity compared to mock Pmel-1 (3**B**, 3 **C**, 3**D**). E: Kaplan-Meier survival plot of mice treated as described above.

A comparable set of experiments is shown in Figure 3 in which DN Pmel-1 or mock Pmel-1 were administered to B16 tumor-bearing mice that then received IL-2, gp100_25-33_/DC, both or neither supporting intervention. In Figure 3A, Pmel-1 were administered to conditioned mice with established B16 tumors; the DN group delayed tumor outgrowth to day 60 with neither IL-2 nor DC vaccine. The administration of IL-2 (3B), gp100_25-33_/DC (3 C) or IL-2/DC (3D) clearly enhances the antitumor activity of both mock and DN Pmel-1. DN Pmel-1 cells have superior anti-tumor biology *in vivo* compared to mock transduced Pmel in all groups except those animals receiving IL-2/DC in which tumor suppression for all Pmel treated mice extends to day 60. These data are represented in Kaplan-Meier plots in Figure 3E where a clear pattern emerges. Control mice all die by day 24. Only 11% of mock-transduced Pmel treated mice survived to day 120 whereas 53% of DN-transduced Pmel mice were still alive.

### Tumor infiltrating DN TGFβ pmel-1 T cells remain activated in the tumor microenvironment

**Figure 4:**
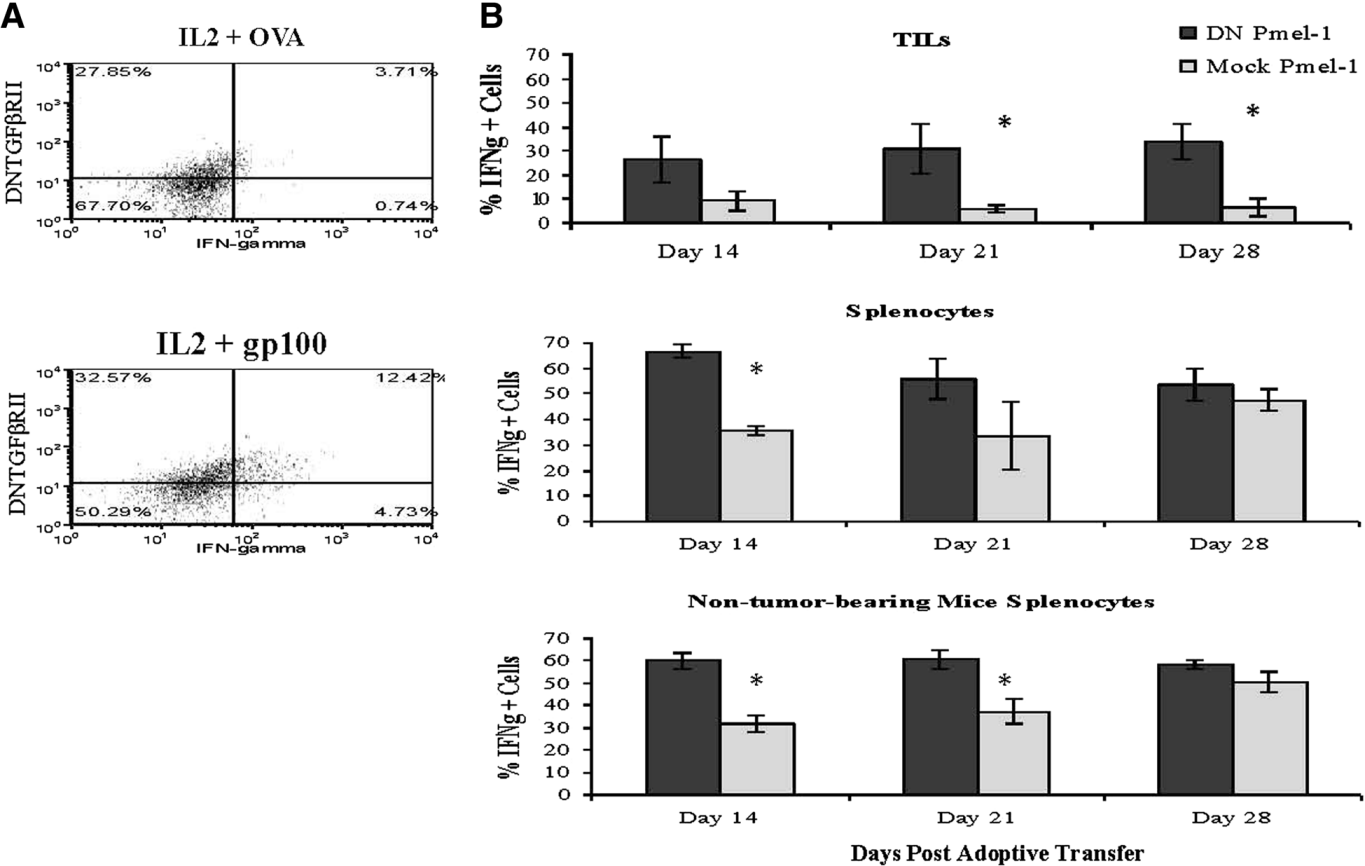
**TGFβ insensitive Pmel-1 remain activated in immunosuppressive tumor microenvironment.** On day 14, 21, and 28 post co-adoptive transfer of DN and mock transduced Pmel-1 to B16 tumor-bearing and non tumor-bearing mice, splenocytes and tumor-infiltrating lymphocytes (TILs) of treated mice were analyzed for IFN-γ production by intracellular cytokine staining. Between 2x10^4^-3x10^5^ TILs and 1x10^7^-6x10^7^ splenocytes were collected on each day of experimentation. Figure 4**A** shows representative dot plots of TILs harvested from DN/mock mixture pmel-1 injected mice stimulated with either IL2 + control OVA peptide or IL2 + melanoma specific gp100_25-33_ peptide. About 25-35% of DN TGFβ RII tumor-infiltrating lymphocytes were actively secreting IFN-γ upon re-stimulation compared to about 10% of mock-transduced TILs (4**B**). In both tumor-bearing and non tumor-bearing mice (all mice received DC vaccinations), a higher % of DN Pmel-1 splenocytes produce IFN-γ on day 14 and 21 post adoptive transfer compared to mock Pmel-1 splenocytes (4**B**). Experiment was performed twice with similar results.

DN and mock-transduced Pmel-1 were co-adoptively transferred to B16 tumor-bearing and non tumor-bearing mice; we wanted to compare their relative expansion, tumor infiltration, and activation *in vivo*. Tumor infiltrating and spleen repopulating Pmel-1 were retrieved at various intervals (days 14, 21, 28 post ACT) and evaluated for relative number and percent of cells producing IFN-γ (by intracellular cytokine staining). There was not a reproducible preferential expansion or infiltration of either Pmel-1 population in the tumor or spleen, based on total cells counts analyzed from the harvested tissues (Additional file 1: Figure S1). However, in replicate experiments, a higher percentage of DN Pmel-1 retrieved from B16 tumors stained for IFN-γ. As shown in Figure 4B, the differences were very significant at all time points for tumor infiltrating Pmel-1, with DN population having a several fold higher percentage of IFN-γ production. Spleen repopulation with the DN Pmel-1/mock Pmel-1 co-adoptive transfer also demonstrated higher IFN-γ production among DN Pmel-1 on days 14 and 21 but not 28, in both naïve and B16 tumor-bearing mice, and to a comparable degree. Analysis of spleen repopulation suggests that DN Pmel-1, in general, can maintain a higher level of Th1 cytokine production upon adoptive transfer into conditioned hosts. A remarkable comparison is between spleen and tumor-infiltrating Pmel-1, and the profound suppression of cytokine production in the latter population. These findings suggest that DN Pmel are functionally more active within the tumor microenvironment.

## Discussion

We have shown that TCR-transgenic T cells transduced with a TGFβ dominant-negative receptor have superior anti-tumor activity against B16 melanoma in a model of adoptive cell therapy. This work extends the findings of several other groups using an identical DN receptor in a variety of models of auto-immunity and tumor immunity. The novelty of this confirmatory report is the use of TCR-transgenic T cells that recognize a self tumor antigen in a commonly used preclinical animal model. DN TGFβ-transduced Pmel-1, on a cell for cell basis, were more than ten-fold more potent in mediating tumor regression of established tumors. We observed complete and durable regression in some mice receiving DN Pmel-1 with neither IL-2 nor DC vaccine support. Pmel-1 CD8 T cells retrieved from B16 tumor microenvironment are immunosuppressed; fewer than 10% express IFN-γ by intracellular cytokine staining. In contrast, 25-35% of tumor-infiltrating DN Pmel-1 continued to produce IFN-γ, suggesting that these engineered T cells are more biologically active in the tumor microenvironment.

TGFβ is a pleiotropic cytokine which plays a significant role in various cellular processes including cellular proliferation, differentiation, and activation [8,25]. Multiple studies have demonstrated a direct correlation between TGFβ expression and tumor growth in melanoma and various other types of cancer [26]. TGFβ has an anti-proliferative effect and is considered to be a tumor suppressor during early stages of tumor development; however, during later stages of carcinogenesis, large amounts of TGFβ are secreted from many tumor types and have been implicated in immune evasion, mainly through negative regulation of immune effector function [27].

The DN TGFβ RII transgene has been demonstrated to be an effective strategy to circumvent the inhibitory effect of TGFβ. Upon TGFβ binding to the extracellular region of the TGFβ receptor complex, intracellular kinase domain phosphorylates SMAD2 and SMAD3 protein, which translocate to the nucleus along with SMAD4, forming a complex that regulates gene expression. DN TGFβ receptor II lacks the intracellular kinase domain thus inhibiting intracellular signaling upon TGFβ binding. Mice with T cells that express this DN receptor as a transgene could reject various murine tumors including lymphoma and melanoma [16]. These elegant experiments have shown enhanced cross-presentation of tumor antigen and expansion of antigen-reactive T cells. Apart from inhibiting T cell effector function, TGFβ has an anti-proliferative effect. *In vitro*, exogenous TGFβ inhibits proliferation of wild-type T cells while DN transduced T cells retain proliferative and lytic function [12-14]. These TGFβ DN transgenic mice, at 12 wks, develop lethal multi-organ autoimmune disease [8,9]. This finding underscores the key role of TGFβ in Treg maintenance of peripheral tolerance.

Several reports have demonstrated the ability of adoptively transferred DN CD8+ T cells to mount potent anti-tumor response against solid tumors. The anti-tumor response is mainly attributed to increased immune effector function of adoptively transferred T cells. Whereas wild-type or TGFβ sensitive T cells are rendered anergic by tumor secreting TGFβ, T cells that are insensitive to TGFβ signaling retain their cytolytic function and demonstrate the ability to produce IFN-γ as well as granule exocytosis [12-15].

Natural killer and dendritic cells are also negatively regulated by TGFβ. TGFβ inhibits expression of MHC II, costimulatory molecules, and cytokine production by dendritic cells. Tian et al. demonstrated that blockade of TGFβ signaling in dendritic cells leads to enhanced anti-tumor activity in a murine renal carcinoma vaccine model [28].

Tumor-secreted TGFβ not only affects T cells through direct inactivation and growth inhibition, but also plays a significant role in the maintenance and function of regulatory T cells [29-31]. It is well established that TGFβ promotes generation of induced regulatory T cells upon TCR stimulation and supports their survival in the periphery. Chen et al. showed that regulatory T cell dependent inhibition of tumor-specific CD8+ T cell mediated cytotoxicity requires the expression of TGFβ receptor, as CD8+ T cells incapable of TGF β signaling were resistant to suppression by regulatory T cells [10].

TGFβDNRII-transduced T cells are currently being used in clinical trials for Epstein-Barr virus (EBV)-associated malignancies Hodgkin and non-Hodgkin lymphoma [13,14]. In preclinical models, DNtransduced EBV-specific T cells have a functional advantage over unmodified T cells: they were resistant to TGFβ-mediated inhibition of proliferation cytolytic activity and mediated superior antitumor activity in a murine tumor model.

In summary, we demonstrate that antigen-specific T cells rendered insensitive to TGFβ through retroviral transduction of a DN TGFβ receptor II gene mount a more effective anti-tumor response to B16 melanoma, most likely due to the ability of TGFβ insensitive T cells to remain active in the tumor microenvironment. Numerous studies have confirmed the presence of tumor infiltrating lymphocytes in resected cancer; however, these CD8+ T cells are functionally anergic when analyzed ex vivo [32]. Introducing a DN TGFβ RII gene into adoptively transferred T cells may prove to be an effective strategy against tumor-mediated inactivation of infiltrating lymphocytes.

## Endnote

This work was supported by R01 CA129816, P01 CA132681, the Keck Foundation, and the Joy and Jerry Monkarsh Research Fund.

## Abbreviations

TGFβ: Transforming growth factor beta; IL2: Interleukin 2; TILs: Tumor-infiltrating lymphocytes; DC: Dendritic cells; TCR: T cell receptor.

## Competing interest

The authors declare that they have no competing interests.

## Authors’ contributions

JQ, YW, DV, LF, AJ and RK conducted animal experiments and analyses, contributed to experimental design and writing. WM, TC and JE contributed to experimental design and writing. All authors read and approved the final manuscript.

## Supplementary Material

Additional file 1 **Figure S1.** Absolute cell counts of MOCK Pmel-1 and DN Pmel-1 cells in the spleen and in the tumor in the conditions described in Figure 4. Click here for file
