# Supplementary figures and images for: T cell receptor (TCR)-transgenic CD8 lymphocytes rendered insensitive to transforming growth factor beta (TGFβ) signaling mediate superior tumor regression in an animal model of adoptive cell therapy
Source: J Transl Med. 2012 Jun 19;10:127. doi: 10.1186/1479-5876-10-127 (PMC3507675; doi:10.1186/1479-5876-10-127)

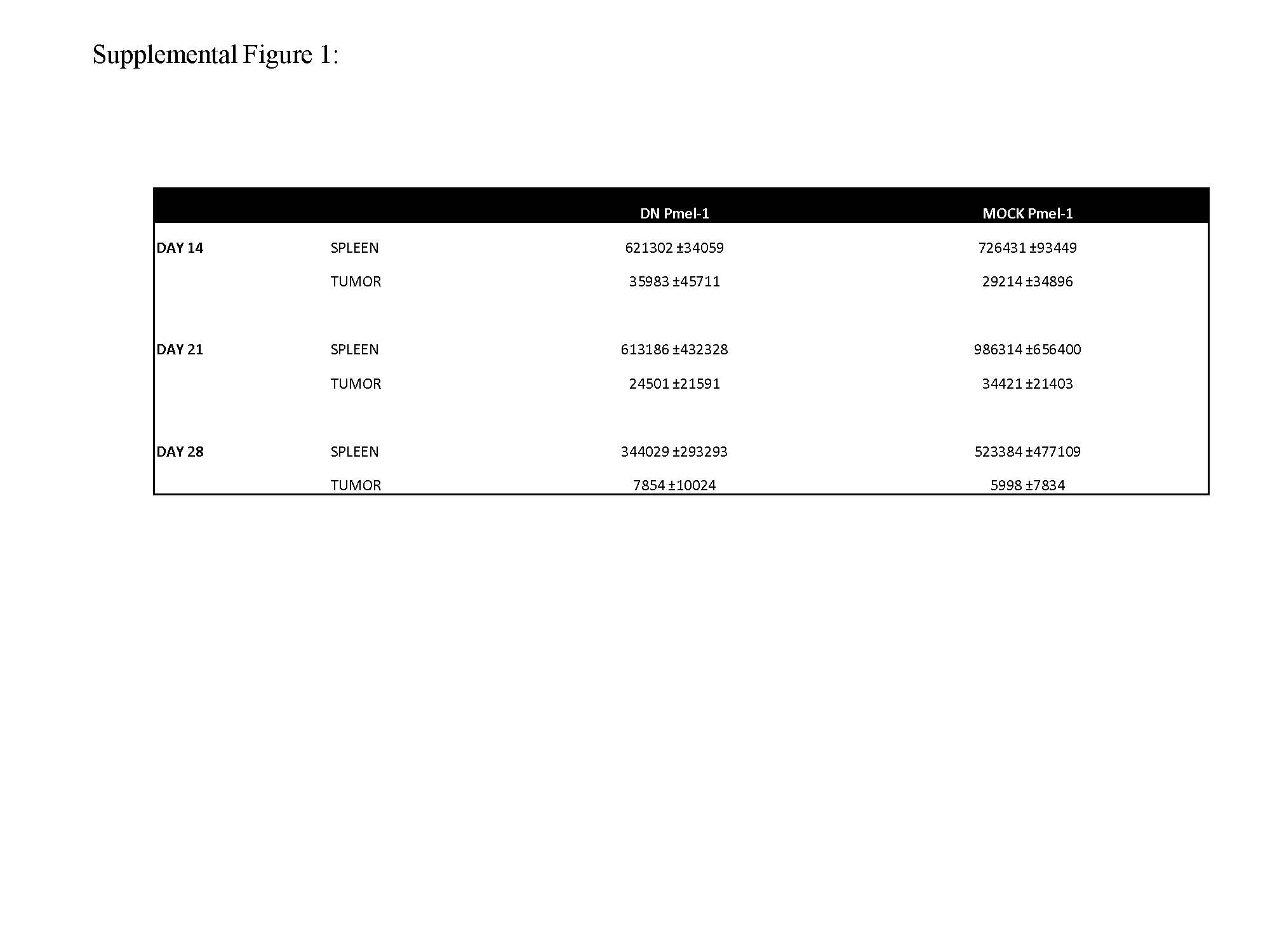

Supplement: Additional file 1 — Figure S1. Absolute cell counts of MOCK Pmel-1 and DN Pmel-1 cells in the spleen and in the tumor in the conditions described in Figure 4. [file 1479-5876-10-127-S1.jpeg]
